# Endometriosis in clear cell and endometrioid carcinoma ovary: its impact on clinicopathological characteristics and survival outcomes

**DOI:** 10.3332/ecancer.2023.1591

**Published:** 2023-08-21

**Authors:** Mythili Kundur, Priya Bhati, Burde Kaustubh Girish, VS Sheejamol, Indu R Nair, Keechilat Pavithran, Anupama Rajanbabu

**Affiliations:** 1 Department of Gynaecological Oncology, Amrita Institute of Medical Sciences, Kochi 682024, India; 2 Department of Biostatistics, Amrita Institute of Medical Sciences, Kochi 682024, India; 3 Department of Pathology, Amrita Institute of Medical Sciences, Kochi 682024, India; 4 Department of Medical Oncology, Amrita Institute of Medical Sciences, Kochi 682024, India; 5 James Cook University Hospital, Middlesbrough TS4 3BW, UK; a https://orcid.org/0000-0002-2885-8098

**Keywords:** endometriosis associated ovarian cancer, clear cell ovarian cancer, endometrioid ovarian cancer

## Abstract

**Background:**

Malignant transformation in endometriosis was first described by Sampson in 1925. There is now sufficient evidence of its association specifically with endometrioid (EOC) and clear cell ovarian cancer (CCOC). Whether endometriosis-associated ovarian cancer (EAOC) is a distinct clinicopathological entity from non-endometriosis-associated ovarian cancer (NEAOC) remains uncertain.

**Objectives:**

This study aimed to assess the impact of endometriosis on clinical characteristics and survival outcomes in EOC and CCOC.

**Methods:**

This is a retrospective single-institution analysis of patients diagnosed with CCOC AND EOC between 2010 and 2021. Demographic and clinical presentation data were obtained from medical records. Patients were followed up till March 2023. Statistical analysis was done using the IBM SPSS Statistics 20 Windows.

**Results:**

Of the 77 cases of CCOC and EOC ovary, 38 had histopathologically proven endometriosis. There was no difference in age (51.62 and 50.05 years, respectively), body mass index, parity, menopausal status and CA 125 levels at presentation. Ascites was more frequent in the absence of endometriosis (30% versus 8.1%, *p* = 0.015). However, this did not translate to a statistical difference in the stage, with the majority presenting in the early stage. (94% versus 83%). All 78 patients underwent primary cytoreduction with equal rates of optimal resection.

There was no difference in the mean disease-free interval between EAOC and NEAOC (107.6 and 109.4 months, p 0.484). Recurrences were predominantly pelvic in both groups. The disease-specific survival was 111.7 and 120.1 months, respectively, with and without endometriosis. This was however not statistically significant (*p* 0.751).

**Conclusion:**

In the Indian population, endometriosis did not have any impact on the age at presentation, CA 125 levels, stage of the disease and survival outcomes in EOC and CCOC ovary.

## Introduction

Endometriosis, although a non-neoplastic gynaecological condition, confers a 1.2–1.8 times higher risk of ovarian malignancy [1–3]. First described by Sampson [4], studies have now demonstrated pathological evidence of endometriotic lesions progressing to malignancy, with an entity called atypical endometriosis being a step in the transition [4, 3]. Alterations in the microenvironment and oxidative stress in endometriotic lesions are hypothesised to trigger carcinogenesis. It is most often associated with a clear cell or endometrioid subtypes of ovarian cancer, the risk of which is increased 2–3 fold in the presence of endometriosis [1, 5–7].

Whether endometriosis-associated ovarian cancer (EAOC) is a distinct clinicopathological entity from non-endometriosis-associated ovarian cancer (NEAOC) remains uncertain. While some studies have shown EAOC to occur at an earlier age, present at an early stage and have a more favourable prognosis, others have shown no such association [8–12]. Most data are retrospective, and the majority of the studies were conducted in South East Asian countries, where the prevalence of these subtypes is higher [1].

This study aimed to assess the impact of endometriosis on clinical characteristics and survival outcomes in Endometrioid and clear cell ovarian carcinoma (EOC, CCOC) in the Indian population.

## Methods

This is a retrospective single-institution analysis conducted at Amrita Institute of Medical Sciences, Kochi. All patients diagnosed with clear cell or endometrioid subtypes of ovarian cancer between 2010 and 2021 were included in the study. Exclusion criteria were patients lost to follow-up and those who did not complete treatment.

Demographic data, clinical presentation, tumour markers (CEA, CA125), FIGO stage, pathological subtype and treatment details were obtained from the hospital's electronic medical records.

The presence of endometriosis on histopathological examination of the surgical specimen was mandatory to label as EAOC. Patients were followed up in the hospital in person or telephonically till March 2023.

Disease-free interval was defined as the time from detection of the disease to the first sign of recurrence, either clinical, biochemical or radiological. Overall survival was defined as the interval between disease detection to death. Patients who died due to reasons other than malignancy were excluded from the calculation of disease-specific survival.

Statistical analysis was done using the IBM SPSS Statistics 20 Windows (SPSS Inc., Chicago, USA). The results are given in mean±SD for all the continuous variables and in frequency (percentage) for categorical variables. The Pearson chi-square test was used for finding the association between two categorical variables. Kaplan Meier curve and log-rank test were used to compare the average survival time and disease-free survival between patients with and without endometriosis. A *p*-value of <0.05 was considered statistically significant. All tests of statistical significance were two-tailed.

## Results

Between 2010 and 2021, 77 eligible cases were identified, of which 35 were CCOC, 41 were EOC and 2 were of mixed histology; 38 of the 77 cases had pathologically proven endometriosis, equally distributed to both subtypes as shown in Table 1. No patients were lost to follow-up.

The clinical and disease characteristics of the two groups are presented in Table 2. Both groups were demographically similar. The mean ages were comparable between the groups, 51.62 and 50.05 years, respectively. Both EAOC and NEAOC occurred predominantly in parous women. Abdominal pain and distension were the most common presenting symptoms across both groups. Both groups had a comparable elevation in CA 125, while CEA was within normal limits in all the patients.

Ascites was significantly more common amongst patients without endometriosis. This, however, did not translate to a statistical difference in the stage and both early and advanced stages were equally associated with ascites. A higher number of patients with EAOC had early-stage disease (94.6% and 79.5%) and the percentage of patients with advanced disease was more with NEAOC (5.4% and 20.5%, respectively). However, this difference was not statistically significant (*p* 0.108). Majority of the patients had a unilateral ovarian mass at diagnosis regardless of endometriosis.

EOC was predominantly low grade in both groups (91% of EAOC and 77% of NEAOC, respectively, *p* 0.568). Amongst those with EOC, three patients in each group had a synchronous endometrial malignancy. Additionally, one patient with NEAOC had coexisting atypical endometrial hyperplasia.

Only 53 of the 77 patients had immunohistochemistry (IHC) reports available. Amongst them, there was no difference in the expression of estrogen receptor (ER) (64% versus 72%, *p* 0.544), progesterone receptor (PR) (59% versus 50%, *p* 0.821) or p53 (16.4% versus 8.7%, *p* 0.666) between EAOC and NEAOC.

All patients underwent primary cytoreductive surgery. 35 patients with EAOC (94.6%) and 37 with NEAOC (92.5%) had complete cytoreduction, while 1 from each group (2.7% and 2.5%, respectively) had optimal cytoreduction with less 1 cm residual disease. Two patients had incomplete staging surgery, one due to a frozen pelvis and other for presumed benign disease.

About 73% of EAOC and 82.5% of NEAOC received adjuvant chemotherapy. The most frequent regimen was 3 weekly Paclitaxel and Carboplatin, of which the majority completed six cycles.

After a median follow-up of 61 months, there was no statistically significant difference in the disease-free interval and overall survival between EAOC and NEAOC.

**Figure 1.**
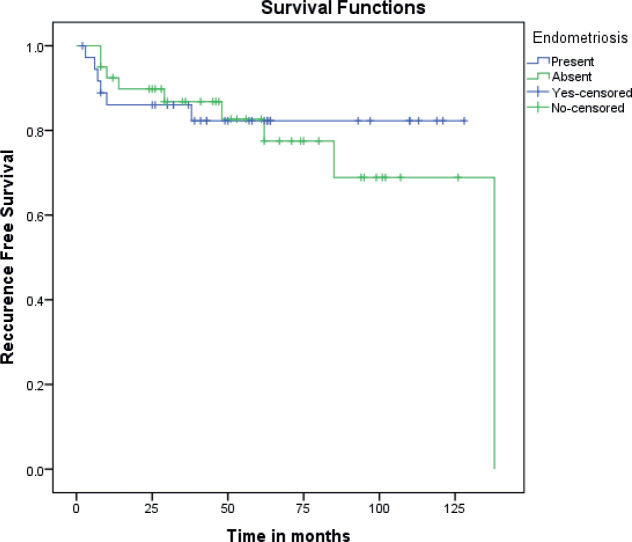
Kaplan Meir curves comparing recurrence-free survival between EAOC and NEAOC.

The mean disease-free interval was 107.6 and 109.4 months in those with and without endometriosis respectively (*p* 0.484). This translated to 5 years disease-free survival of 82.3% and 82.7% (Figure 1).

Two patients had progressive disease. One with EAOC and stage III disease, and the other with NEAOC and stage IV disease.

**Figure 2.**
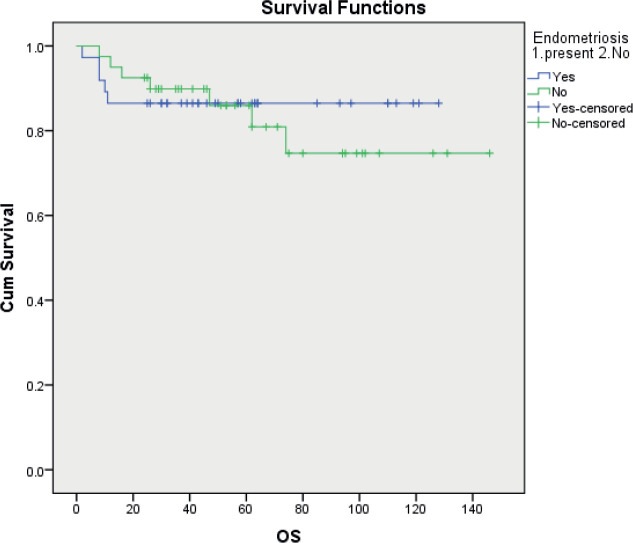
Kaplan Meir curves comparing disease-specific overall survival between EAOC and NEAOC.

Overall survival was similar in the two groups with a 5 years OS of 86.5% and 81.9% for EAOC and NEAOC, respectively (111.7 and 116.7 months, *p* 0.556) (Figure 2). Three patients died of causes unrelated to the malignancy. The disease-specific survival was 111.7 and 120.1 months, respectively, with and without endometriosis. This difference did not reach statistical significance (*p* 0.751).

On univariate analysis, the stage of the disease was the only significant prognosticator of disease-free and overall survival.

## Discussion

Endometriosis, despite being a non-neoplastic disease, often behaves like a malignancy, with a tendency of tissue invasion and spread. Additionally, an altered microenvironment and abnormal immunoregulation around endometriotic lesions confer a predilection for malignant transformation.

In the present study, 48% of CCOC and EOC were associated with endometriosis. Studies have shown a wide variation in this regard. Zhou *et al* [8] had an incidence of 54%, comparable to the findings of this study. Meanwhile, Barreta *et al* [9] found 80.0% of CCOC and EOC to be associated with endometriosis.

The age at diagnosis in the present study was 51 and 50 years in EAOC and NEAOC, respectively, and did not differ significantly between the groups. This was comparable to studies in other ethnicities, except for Paik *et al* [11] who observed an earlier age of onset in EAOC. The same studies reported an earlier age of onset in EOC compared to CCOC, regardless of the presence of endometriosis[8, 9]. The present study did not analyse the differences between the subtypes of ovarian cancer.

While Barreta *et al* [9] had predominantly nulliparous and premenopausal women in their population of EOC and CCOC, a larger proportion of our population was parous. Neither parity nor menopausal status was significantly different with regard to endometriosis.

CA 125 was significantly above normal in most patients, despite 85% of all patients having early-stage disease. This elevation was unrelated to the presence or absence of endometriosis. Barreta *et al* [9] had similarly elevated levels in their study. These findings are contrary to the traditional belief that CA 125 is unreliable in non-high-grade serous variants of ovarian cancer [6].

Majority of patients with both EAOC and NEAOC had stage I or II disease at diagnosis, similar to Cai *et al* [12] findings and two other similar studies from Korea [10, 11]. Although not statistically significant, the proportion of patients with stage III and IV cancer was higher for NEAOC. On the contrary, other studies in the Asian population and one in the Brazilian population found more evidence of endometriosis in the early stages [8, 9]. It was observed that endometriotic tissue became increasingly scarce or absent with advanced stages, likely due to complete malignant transformation or infiltration by the tumour. This may lead to underestimation of endometriosis in advanced cancers, which have a poorer prognosis. To overcome this, they speculated that patients with a history of past endometriosis should be considered to have EAOC, as an absence of endometriosis in pathological specimens does not mean it was absent prior to malignant transformation. Symptoms of endometriosis such as dysmenorrhoea likely prompt patients to seek medical attention. This is another reason for early-stage presentation in endometriosis patients, whereas NEAOC may become symptomatic only when advanced [12]. 

ER, PR IHC was not uniformly available for all patients in the current study, but amongst available cases, there was no difference between the groups. Zhou [8] demonstrated that ER positivity was higher in EOC than in CCOC but did not correlate it to endometriosis. Studies have suggested that loss of ER expression might be a key event in making CCOC aggressive [12, 13].

It is known that endometrioid and clear cell cancers have a favourable prognosis when detected early and are aggressive when advanced. In a study by Erzen *et al* [14] endometriosis was an important prognosticator, associated with a longer time to recurrence and significantly improved survival. However, the present study found no impact of endometriosis on the recurrence of the disease or overall survival. Our findings resonated with the study by Barreta *et al*. [9] Cai *et al* [12] studied EOC and CCOC only in young (<40 years) Chinese patients and had similar conclusions.

Zhou *et al* [8] in a 21-year-old retrospective analysis of 211 patients found no association between survival outcomes and endometriosis. Similar to the present study, they also identified the FIGO stage to be the most important prognosticator.

Barreta *et al* [9] also showed stages III and IV to be the only prognostic factor of importance, that too only in CCOC, regardless of the presence of endometriosis. Ju *et al* [10] in their multivariate analysis established residual disease after surgery as the only prognosticator of survival. Although it is a precursor for cancer, endometriosis does not seem to impact the course of the disease once malignant transformation has occurred [18].

EAOC is defined variably in studies. Some considered the presence of endometriosis within the surgical specimen sufficient, similar to the present study [11]. However, others had more stringent criteria such as evidence of malignant transformation within the endometriotic glands, or even employed immunohistochemical markers such as CD10 to identify EAOCs [15, 16]. The mere presence of adjacent endometriosis may not confer the distinct biology of EAOC as a malignant transformation of the endometriotic glands themselves. This is reiterated by data showing the presence of molecular alterations such as ARID1A mutations typical of EAOC in contiguous atypical endometriosis but not in adjacent endometriotic lesions [17]. Perhaps the positive impact of endometriosis on prognosis was significant in some studies such as by Lu *et al* [16] as they systematically identified cancer arising from endometriosis based on strict criteria and excluded those merely who merely had coexisting endometriosis. However, markers such as IHC come with cost constraints, especially if performed on all tumour sections. It can be done only in areas where there is a morphological suspicion of residual endometrial-type stroma.

Similä-Maarala *et al* [19] found that endometrial cancer-based molecular classification was applicable to EAOC and was particularly marked in CCOC. In both EOC and CCOC, no specific molecular profile constituted the largest group. P53 abnormality conferred a poor prognosis in both groups while DNA polymerase epsilon mutation was associated with a favourable prognosis. Mismatch repair deficiency was associated with excellent prognosis in CCOC and not in EOC. The application of molecular classification may thus aid in prognosticating this group of patients [19].

EOC and CCOC, while being distinct in their behaviour from the more prevalent serous subtype, also different from each other in their behaviour. This is evidenced by studies showing higher age and advanced stage at diagnosis along with poorer prognosis with CCOC. This raises the question of how two distinct entities arise from a common risk factor-endometriosis. Recent data employing proteomic studies suggest that EOC arises from the secretory cells of the ectopic endometrium while CCOC originates from ciliated cells [20]. Whether the clinicopathological features and prognosis are related to the histological subtype, namely, CCOC or EOC, rather than the presence or absence of endometriosis is worthy of exploration.

Small sample size was a limitation of this study. It did not allow for analysing the impact of endometriosis on CCOC and EOC separately. The larger number of advanced-stage NEAOC did not reach statistical significance in the present study. Perhaps a larger sample would clarify if the absence of endometriosis in fact predisposes to advanced malignancy in these two subtypes.

## Conclusion

In the Indian population, endometriosis did not have any impact on the age at presentation, CA 125 levels, stage of the disease and survival outcomes in endometrioid and clear cell cancer of the ovary.

Due to the relatively low incidence of EOC and CCOC, larger multi-centre studies with clearly defined criteria to identify a cancer as EAOC are required to shed light on whether it is the histological subtype or the association with endometriosis that impacts the biological behaviour and survival outcomes.

## Abbreviations

EAOC: Endometriosis-associated ovarian cancer; EOC: Endometrioid ovarian cancer; CCOC: Clear cell ovarian cancer; IHC: Immunohistochemistry; NEAOC: Non-endometriosis-associated ovarian cancer.

## Conflicts of interest

None.

## Funding

None.

## Figures and Tables

**Table 1.** Distribution of cases.

Histopathology	Endometriosis - present		Endometriosis - absent		% Total
	Frequency	%	Frequency	%	
Endometrioid	19	51.4	21	52.5	51.9
Clear cell	16	43.2	19	47.5	45.45
Mixed histology	2	5.4	0	0	2.59
Total	37	100	40	100	100

**Table 2.** Comparison of demographic and clinical features.

	Endometriosis- present	Endometriosis- absent	*p* value
	(*n* = 38)	(*n* = 39)	
Age at diagnosis (Mean ± SD)	51.62 ± 10.433	50.05 ± 13.687	0.575
Body mass index (Mean ± SD)	25.10 ± 3.57	25.08 ± 3.606	0.975
Parity			
Nulliparous	8 (21.6%)	11 (27.5%)	0.55
Parous	29(78.4%)	29 (72.5%)	
Menopausal status (*n* (%))			
Premenopausal	14 (37.8%)	19 (47.5%)	0.392
Postmenopausal	23 (62.2%)	21 (52.5%)	
Presenting complaints (*n* (%))			
Pain Abdomen	14 (37.85%)	18 (45%)	0.963
Distension	9 (24.3%)	10 (25%)	
Mass	1 (2.7%)	1 (2.5%)	
Menstrual symptoms	7 (18.9%)	6 (15%)	
Non specific	6 (16.2%)	5 (12.5%)	
Ovarian mass (*n* (%))			
Unilateral	34 (91.9%)	39 (97.5%)	0.268
Bilateral	3 (8.1%)	1 (2.5%)	
Ascites (*n* (%))	3 (8.1%)	3 (8.1%)	0.015
FIGO stage (*n* (%))			
Early (stage I and II)	35 (94.6%)	31 (79.5%)	0.108
Advanced (stage III and IV)	2 (5.4%)	8 (20.5%)	
